# The Improvement Effect of D-Chiro-Inositol and *Ecklonia cava* K. in the Rat Model of Polycystic Ovarian Syndrome

**DOI:** 10.3389/fphar.2022.905191

**Published:** 2022-07-19

**Authors:** Hyun Yang, Sang R. Lee, Seong Lae Jo, Ae-Hyang Kim, Eun-Ryoung Kim, Fan Qu, Eui-Ju Hong, Hye Won Lee

**Affiliations:** ^1^ KM Convergence Research Division, Korea Institute of Oriental Medicine, Daejeon, South Korea; ^2^ College of Veterinary Medicine, Chungnam National University, Daejeon, South Korea; ^3^ Amicogen Inc., Jinju, South Korea; ^4^ Women’s Hospital, School of Medicine, Zhejiang University, Hangzhou, China

**Keywords:** DCI, d-chiro-inositol, ovarian failure, polycystic ovary syndrome, follicle formation, *Ecklonia cava* K.

## Abstract

**Introduction:** Polycystic Ovarian Syndrome (PCOS) is known to be an endocrine state that is characterized by oligomenorrhea, hyperandrogenism, and highly cystic follicles in the ovaries. The use of food ingredients and traditional medicine in Asian countries is well known, and previous studies have shown that *Ecklonia cava* K. [Alariaceae] (EC) is able to alleviate PCOS symptoms. D-Chiro-inositol (DCI) administration in pathologies where steroid biosynthesis is a crucial factor, i.e., PCOS, has provided satisfactory results.

**Methods:** Therefore, we studied the synergistic effects of the two previously known active compounds. In rats with letrozole-induced PCOS, we focused on alternative therapies using EC and/or DCI extracts to alleviate ovarian failure.

**Results:** As a nonsteroidal aromatase inhibitor, letrozole inhibits the conversion of testosterone to estrogen and subsequently causes PCOS. We divided 6-week-old female mice into the following six groups and evaluated them: vehicle, PCOS, PCOS + MET (metformin), PCOS + DCI, PCOS + EC, and PCOS + DCI + EC. In our study, PCOS rats treated with EC and DCI had low serum LH and T levels and low serum levels of inflammatory cytokines such as TNFα and IL-6. These treatments also appeared to regulate the production of factors that affect follicle formation and inflammation in the ovaries.

**Conclusion:** We concluded that EC extract and/or DCI administration influenced aromatase production and reduced LH and T stimulation, and cotreatment with EC and DCI consequently restored ovarian dysfunction or anti-inflammatory responses in rats with PCOS-like symptoms.

## Introduction

Polycystic ovarian syndrome (PCOS) is a women’s endocrine disorder that affects healthy premenopausal women. PCOS patients are generally reproductive women who suffer from abnormal estrous cycles, obesity, hyperandrogenism, and infertility. In PCOS patients, follicle development is interrupted, cysts are formed, and the follicles are eventually depleted. PCOS patients may become naturally pregnant after treatment (Rausch et al., 2009). Diagnostic features include ovarian dysfunction, polycystic ovarian morphology, and hyperandrogenism. Although there is no clear cause of PCOS, the imbalance between endogenous hormones, especially high levels of androgen, can also be seen as being associated with insulin resistance (Trivax and Azziz, 2007), (Bremer, 2010). The main symptoms of PCOS in women of reproductive age with infertility are oligo/menorrhea, hot flushes, vaginal atrophy, and ovulation disorders as other symptoms (Bates and Legro, 2013; Bashir et al., 2018). The causes of PCOS include autoimmune, genetic defects, chemoradiotherapy, ovarian surgery, and environmental factors (Falorni et al., 2014). In addition, PCOS is known to be characterized by elevated levels of high serum LH and a rise in the LH/FSH (follicle-stimulating hormone) ratio (Sahmay et al., 2014).

*Ecklonia cava* K. [Alariaceae] (EC) is a marine brown algae found in the seas and oceans off of Korea, and EC was recorded in an ancient Korean medical book named “Donguibogam.” It has been widely used to treat digestive problems (Donguibogam-Committee, 1999). It is utilized as an herbal therapeutic agent in the form of Seanol and as an extract. The health and chemical benefits of the EC have been previously investigated to reveal its role in dietary supplements and treatment, such as its anti-inflammation, anti-diabetic, and anti-PCOS properties (Yang et al., 2018; Kim et al., 2019). In a previous study by the present authors, EC was found to regulate hormonal disorders of ovarian disease induced by estrogen deficiency through the inhibition of aromatases in rat models.

Furthermore, EC effectively prevented the production of dehydroepiandrosterone and consequently led to a decrease in blood androgen levels (Yang et al., 2018). Myoinositol (MI) and D-chiro-inositol (DCI), two isomers of the family inositolidae, play a decisive role in ovarian physiology (Unfer et al., 2016). In particular, DCI regulates the activity of aromatase and exhibits several effects normally associated with aromatase inhibitors (Lagana and Unfer, 2019). Inhibiting the activity of aromatase enzymes causes androgen to accumulate, resulting in an endocrine imbalance (Daneasa et al., 2016). In PCOS women after failure of ovulation, stimulation of ovulation is still considered the first-line medical approach to improve fertility (Palomba et al., 2004). This treatment is easy to achieve, inexpensive, and does not require monitoring. However, ovulation cannot be achieved without mature follicles for ovulation, and there are many other limitations (Bashir et al., 2018). During the ovarian cycle, the growth and endocrine dynamics of dominant follicles that develop during an unstimulated ovulation cycle with respect to ovulation are not well known.

Chemicals such as antiprogesterone, estradiol valerate, androgens, and letrozole are used to induce PCOS in rodents. These chemicals alter hormone levels in animals and produce features similar to those of human PCOS, cystic ovarian morphology, and hyperandrogenism (Shi and Vine, 2012). Models using letrozole sustained-release formulations are suitable for verifying the therapeutic effect of a drug because the model can be induced in a relatively short time and letrozole easily inhibits natural recovery rather than a medication. We used letrozole-induced PCOS rats as animal models with reproductive and metabolic characteristics similar to human PCOS patients (Kauffman et al., 2015).

To date, there is no reliable treatment for PCOS. PCOS treatment generally focuses on disease management, which relies on symptom relief (Bates and Legro, 2013). The PCOS improvement effects of DCI:MI complex and EC are known in different literatures, respectively (Yang et al., 2018; Bevilacqua et al., 2019). However, the functional differences and synergistic effects of DCI alone, not complex with MI and co-administration with EC, are unknown. In the present study, a rat model of PCOS-like symptoms was used to determine whether EC and/or DCI improve serum hormonal levels, such as LH, FSH, estradiol (E2), and testosterone (T), as well as inflammatory factors related to PCOS symptoms in the blood and ovarian tissue.

## Materials and Methods

### Preparation of *Ecklonia cava* K. Extracts and D-Chiro-Inositol

*Ecklonia cava* K. (EC), D-chiro-inositol (DCI) and EC + DCI provided by Amicogen, Inc. (Jinju, Gyengsangnma-do, South Korea) were used in this experiment. EC collected from Jeju Island, South Korea, was procured from a global herbal medicine store at Cheonnyeon-yagcho (South Korea). Dried EC (1.0 kg) was extracted with water for 3∼4 h, refluxing at 100°C, which was then filtered. To obtain EC powder, the extract was vacuum concentrated and lyophilized in a freeze-dryer. D-chiro-inositol (DCI) was hydrolysis acidic from carob (*Ceratonia siliqua*) bean pod syrup that can be removed methyl from 3-O-methyl-d-chiro-inositol. To remove the impurities, the filtration process and to obtain purified DCI, the vacuum concentration and crystallization process with the addition of ethanol. The resultant material was dried by a vacuum dryer that powder was packed. EC + DCI was mixed at a 1:1 ratio in EC and DCI, respectively.

### Quantitative Analysis of *Ecklonia cava* K. Extracts and D-Chiro-Inositol

For the quantitative analysis of the dieckol, it was purchased from Chem-Norm Biotech Co. Ltd. (Wuhan, China). The EC aqueous extract (1.0 mg) was dissolved in 1 ml of 95% methanol and filtered through a 0.2 μm syringe filter. The samples of EC extract were analyzed by UHPLC-DAD (Shimadzu, Kyoto, Japan). A BRISA LC2 C18 (4.6 × 250 nm, 5 μm) reversed-phased column was used as the analytical column and was retained at 40°C. The mobile phase was composed of distilled water in 0.1% trifluoroacetic acid (A) and acetonitrile in 0.1% trifluoroacetic acid (B), and the gradient system for separation was as follows: 0–3 min, 25% (v/v) B; 3–13 min, 35% (v/v) B; 13–19 min, 90% (v/v) B; 19–25 min, 25% (v/v) B. The injection volume was 20 μL and the flow rate was 1.0 mL/min. The detector wavelength was recorded at 230 nm. The DCI standard (DCI 99%) was procured from Sigma-Aldrich (South Korea). To analyze the content of DCI, the samples were analyzed by HPLC-RI (Shimadzu, Kyoto, Japan). A carbohydrate (4.6 × 250 nm, 4 μm) high performance column was used as the analytical column and was retained at 40°C. The mobile phase was composed of distilled water (A) and acetonitrile (B), as follows: 0–35 min, 85% (v/v) B. The injection volume was 10 μL and the flow rate was 1.0 mL/min.

### Animals and Treatments

Female rats (6-weeks-old, total = 42, group *n* = 7) were obtained from DooYeol Biotech (Seoul, South Korea), and were housed in a specific-pathogen free facility at the Korea Institute of Oriental Medicine under a standard 12 h:12 h light and dark cycle and fed standard chow with water provided ad libitum. Rats were used for the experiment after a week of adaptation, and the experiment was approved by the Ethics Committee of the Korea Institute of Oriental Medicine (approved No. 20-059). A 60-day letrozole sustained-release pellet (1.8 mg/pellet; Innovative Research of America, OH, United States) was used to induce PCOS symptoms, and its implantation into the rats was imbedded subcutaneously under anesthesia (AVERTIN; Sigma-Aldrich, St. Louis, MO, United States). After 2 weeks after insertion of the Letrozole pellet, EC (250 mg/kg) and/or DCI (50 mg/kg) were administered for 2 weeks (p.o.).

### Serum Analysis

Blood samples were collected directly from the inferior vena cava after the end of the experiment, and the serum separation was obtained after centrifugation at 8,000 rpm for 10 min and stored at −70° before being used in the experiment. Luteinizing hormone (LH), Follicle-stimulating hormone (FSH), 17β-Estradiol (E2), and testosterone (T) were measured using an ELISA kit (Enzo Life Science, Inc., Farmingdale, New York, United States). TNFα and IL-6 were measured using an ELISA kit (Abcam, CA, United States). All ELISA kits were used according to the manufacturer’s instructions.

### Reverse Transcription and Real-Time PCR

Total RNA was extracted using TRI Reagent (Molecular Research Center, Cincinnati, OH, United States) following the manufacturer’s instructions. cDNA was synthesized from 1 µg of total RNA with the Thermo Scientific Revert Aid First Strand cDNA Synthesis Kit (Thermo Fisher Scientific, MA, United States). cDNA was amplified by RT-PCR using AmpliTaq Gold DNA polymerase and Quantitative real-time PCR. cDNA was amplified using TaqMan™ Universal PCR Master Mix (Applied Biosystems, CA, United States) with specific probes by the Step One Plus system (Applied Biosystems). All the probes used for Real-Time PCR are as follow: Table 1. All experiments were run in triplicate, and mRNA values were calculated based on the cycle threshold and monitored for an amplification curve.

**TABLE 1:** The list and sequence of primers used for RT-PCR analysis.

No.	Gene	Probes
1	Kitl	Rn01502851_m1
2	Has2	Rn00565774_m1
3	Lhr	Rn00564309_m1
4	Fshr	Rn01648507_m1
5	TNFα	Rn01525859_g1
6	IL-6	Rn01410330_m1
7	COX-2	Rn01483828_m1
8	PPARγ	Rn00440945_m1
8	Rplp0	Rn03302271_gH

### Histological Analysis and Immunohistochemistry

The ovary was fixed in 10% buffered formalin for 48 h and paraffin embedded subsequently. Paraffin embedded tissue sections (5 micrometers) were de-paraffinized, hydrated and stained with haematoxylin and eosin (H&E). The stained slides were examined using the Pannoramic DESK Digital Slide Scanning System (3d Histech, Budapest, Hungary). The section with the largest area was selected for analysis to count the numbers of follicular cysts and antral follicles, thicknesses of the antral follicles. The theca and granulosa layers were measured using by CaseViewer software (3d Histech). To break protein cross-links, the tissue sections were incubated with 0.1 M citrate buffer (pH 6.0) at 95–100°C for 1 h. After blocking with 3% BSA, the slides were incubated with Cyp19a1 antibody (diluted to 1:200) in TBS with 1% BSA (#14528; Cell Signaling Technology; Beverly, MA, United States) at 4°C overnight. Following this, slides were washed and incubated simultaneously with corresponding Alexa-Fluor conjugated secondary antibodies from Life Technologies diluted in TBS with 1% BSA at room temperature for 1 h. After washing, slides were mounted in ProLong Gold antifade reagent with DAPI (Life Technologies, United States) and examined using a DMi8 microscope (Leica Microsystems, Wetzlar, Germany). A vaginal smear was made with PBS solution at the week after letrozole pellet imbedding and before sacrifice. Vaginal smear slides stained with crystal violet (St. Louis, MO 63178, United States) and, after washing, the slides were mounted in glycerol PBS and examined using a DMi8 microscope (Leica Microsystems, Wetzlar, Germany).

### Statistical Analysis

Results for the serum levels of hormones and cytokines, number of follicular cysts, antral follicles, the thickness of cell layers, and transcriptional levels of follicular phase and inflammation-related markers are presented ±SD. Differences between means were obtained by the Student’s t-test, which was performed using Graph Pad Software (GraphPad Inc., San Diego, CA, United States).

## Results

### Quantitative Analysis of Major Compounds in EC + DCI Formular

**FIGURE 1:**
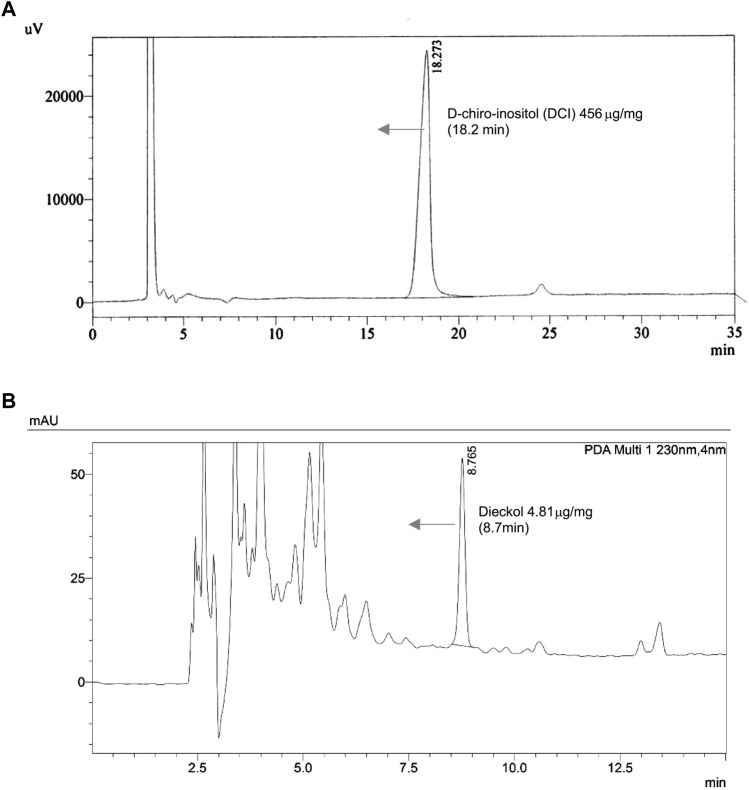
The chromatograms of the **(A)** *d*-chiro-inositol and **(B)** *Ecklonia cava* K. aqueous extract at refractive index (RI) Detector and 230 nm.

Confirmation of the chemical constitutions of the EC + DCI extracts and the two major compounds was carried out by UPLC-PDA and HPLC-RI detector analysis. We finally identified the major compounds. Dieckol was detected at 8.7 min of retention time by 230 nm, its concentration was 4.81 μg/mg. DCI was detected at 18.2 min, its concentration was 456 μg/mg (Figure 1).

### Animal Conditions and Treatments

**FIGURE 2:**
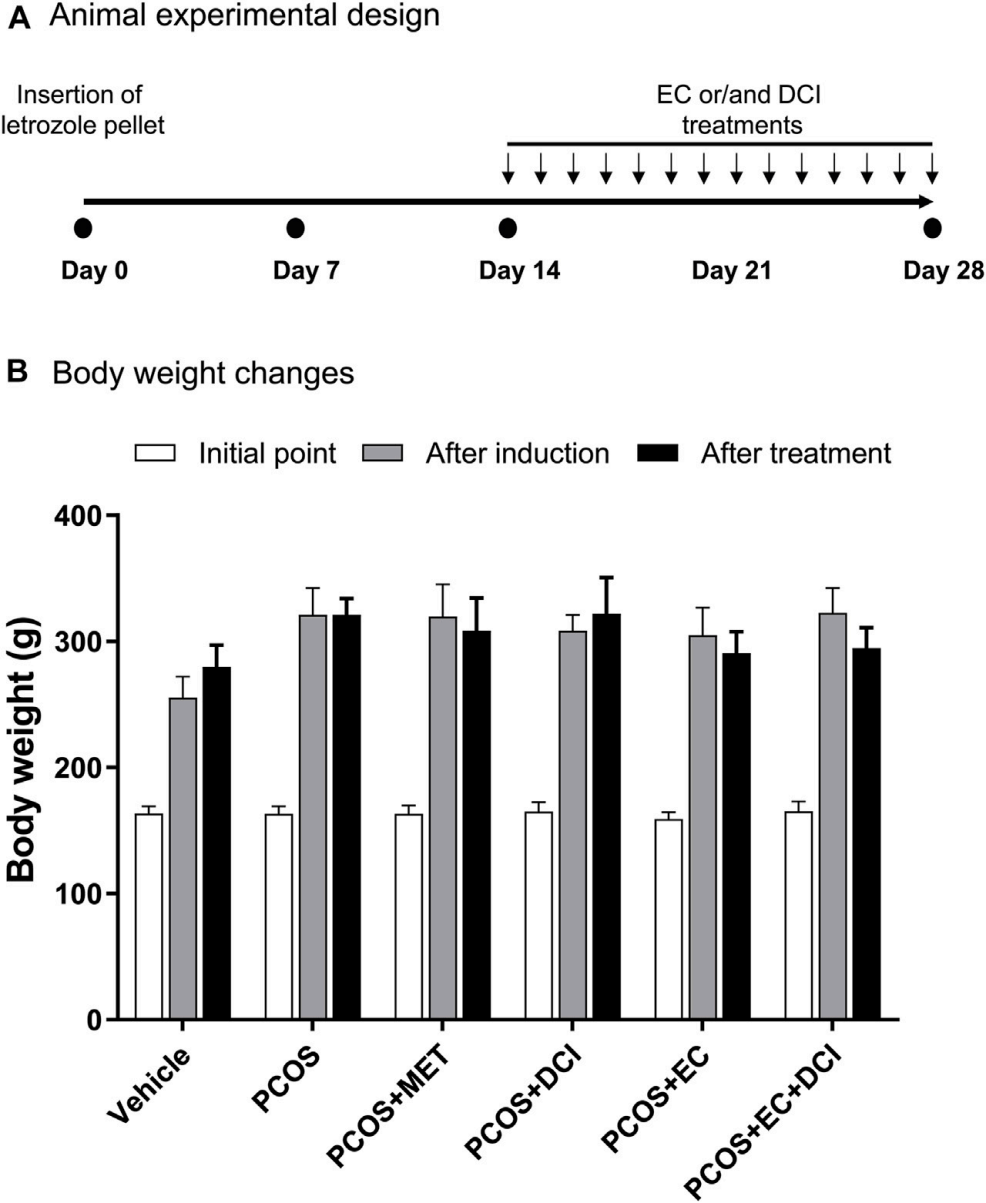
Effect of EC extract DCI on body weight changes. **(A)** Animal experimental design. **(B)** The body weight(s) was measured after the start of the experiment, induction, and drug treatment. ^*^ *p*< 0.05 vs. initial point. MET, metformin; EC, *Ecklonia cava* K.; DCI, d-chiro inositol.

**FIGURE 3:**
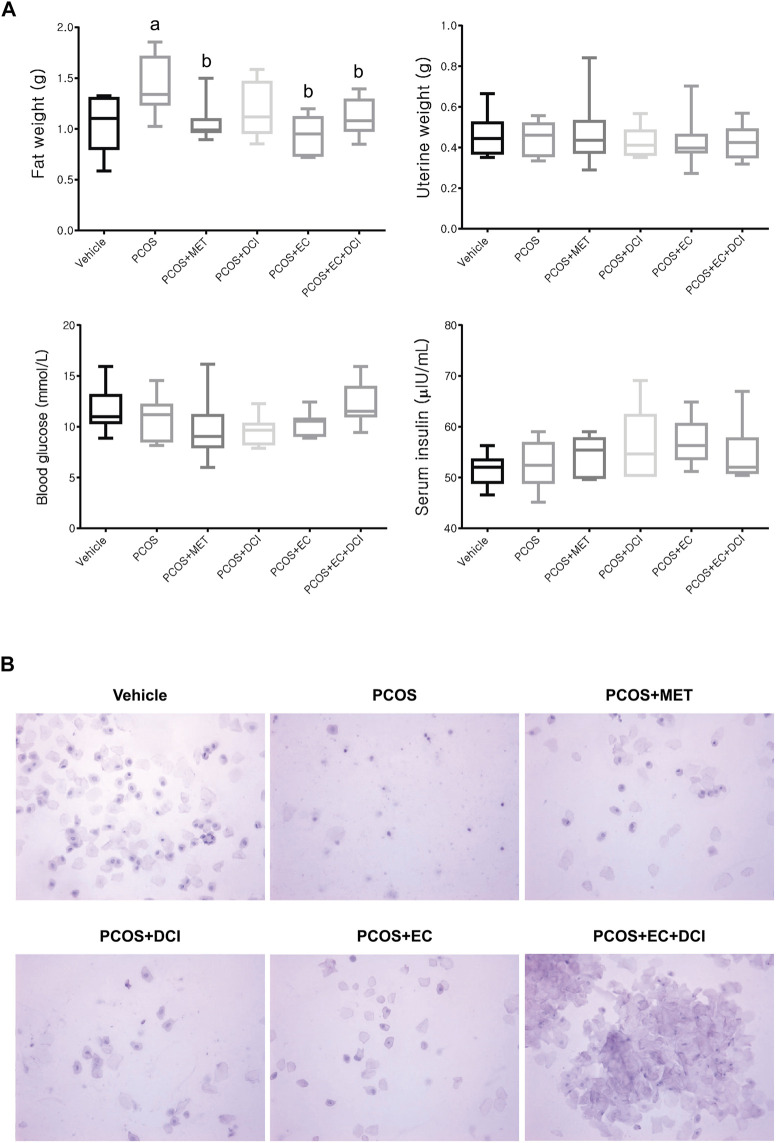
Effect of EC extract DCI on physiological changes. **(A)** The retroperitoneal posterior fat weight, uterine weight, the levels of blood glucose, and serum insulin were measured at the end of the experiment. **(B)** The picture(s) of vaginal smear performed at the end of experiment (×200). MET, metformin; EC, *Ecklonia cava* K.; DCI, *d-chiro* inositol.

To investigate the improvement effects of EC and DCI in the PCOS animal model, we administered EC and/or DCI to letrozole-treated rats for 2 weeks. Letrozole-treated rats were established for 4 weeks using a sustained-release formulation. We measured the weight of rats during the experiment and confirmed the estrous cycle through vaginal cell smears. At the end of the experimental period, the weight gain was similar among the rats implanted with letrozole pellets (Figure 2). In addition, increased retroperitoneal posterior fat in the PCOS group was effectively reduced in the EC and/or DCI treatment groups (Figure 3A). As a result of microscopic examination of vaginal cell smears, white blood cells and basal and parabasal cells were observed in the PCOS group. On the other hand, in the group treated with EC and DCI, more epithelial cells appeared than the number in the PCOS group, and in the group treated with EC and DCI, keratinized cells and nuclear aggregated cells also appeared, as shown in Figure 3B. Our findings suggest that EC and DCI did not ultimately affect weight but inhibited the production of peritoneal posterior fat. It was found that the estrous cycle arrested by letrozole treatment was effectively regulated when EC and DCI were cotreated.

### Effects of the *Ecklonia cava* K. Extract and D-Chiro-Inositol on Serum Hormone and Inflammatory Cytokine Levels

**FIGURE 4:**
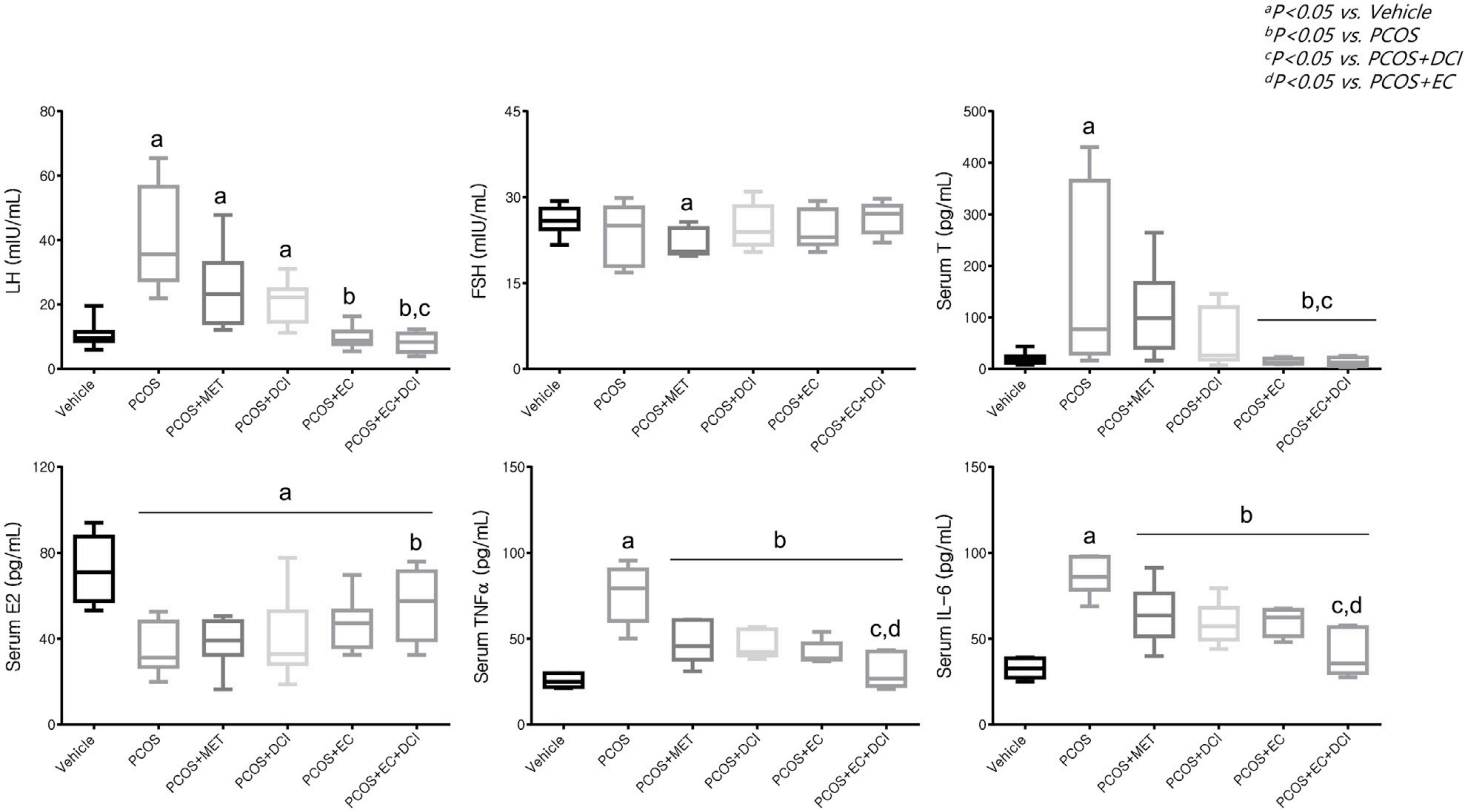
Effect of EC extract and DCI on serum levels in PCOS rats. Serum level(s) of LH, FSH, T E2, TNFα and IL-6 were measured using a competitive enzyme-linked immunosorbent assay (ELISA) kit. All values represent the mean ±SD. ^a^ *p* < 0.05 vs. vehicle, ^b^ *p*< 0.05 vs. PCOS, ^c^ *p*< 0.05 vs. PCOS + DCI, ^d^ *p*< 0.05 vs. PCOS + EC. MET, Metformin; EC, *Ecklonia cava* K.; DCI, *d-chiro* inositol.

Serum FSH levels decreased slightly in the metformin treatment group, but no significant changes were observed among the experimental groups. However, serum LH levels were significantly higher in the PCOS group and tended to be lowered by metformin, EC, and DCI treatment. The lowest serum LH level was observed in the EC and DCI cotreatment groups, which was similar to the LH level in the normal group (Figure 4). In parallel to the serum LH level, the serum T level was significantly higher in the PCOS group. High values were effectively controlled, similarly to normal group values, by EC and DCI cotreatment. Serum E2 levels were found to decrease in all groups treated with letrozole and were significantly increased compared to the PCOS groups only in the group cotreated with EC and DCI (Figure 4). Inflammatory cytokine, TNFα, and IL-6 serum levels were significantly increased in the PCOS group but were effectively decreased in the group treated with EC and/or DCI (Figure 4).

### Effects of the *Ecklonia cava* K. Extract and D-Chiro-Inositol Extract on mRNA Expression Levels

**FIGURE 5:**
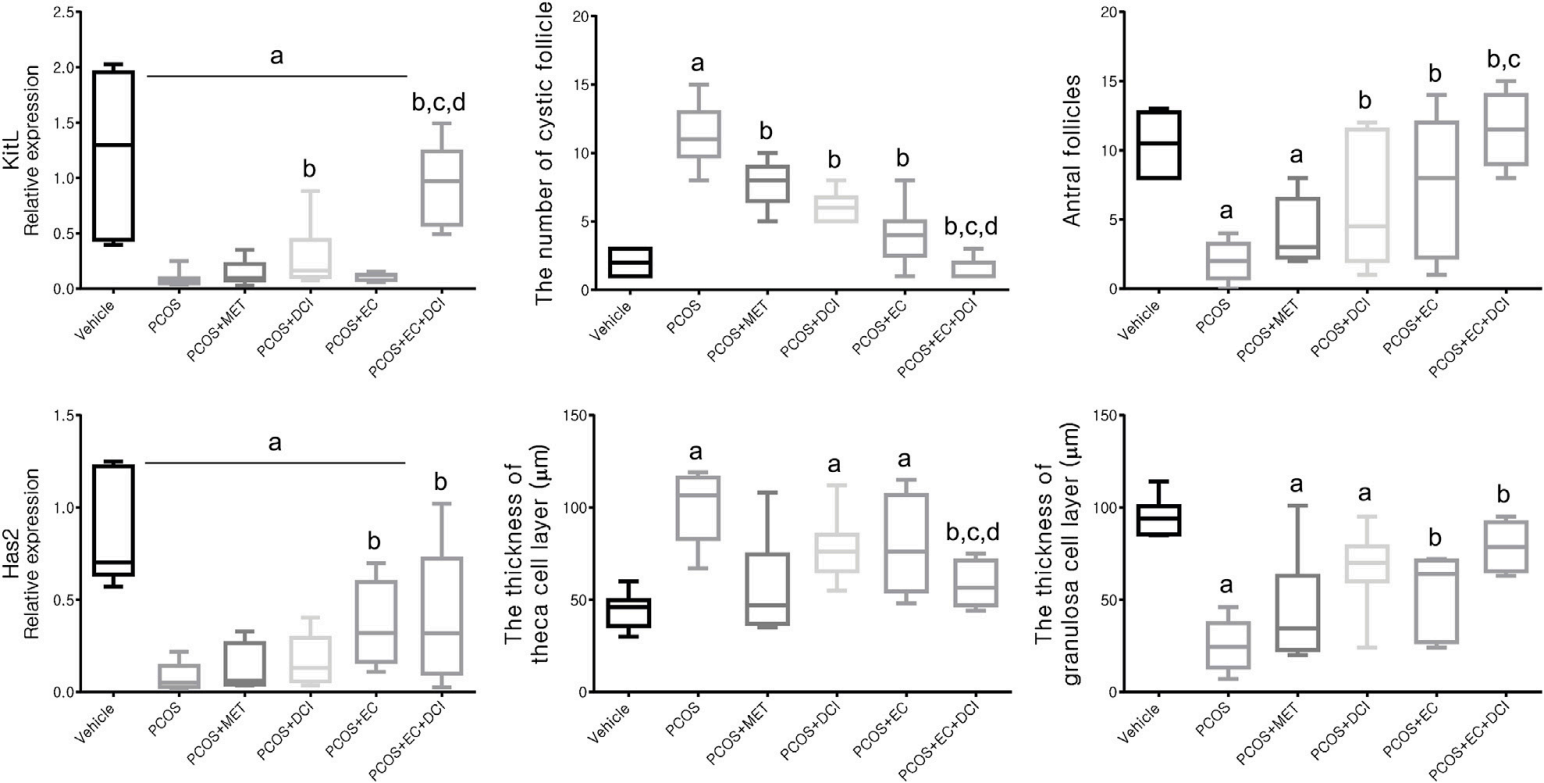
Effect of EC extract and DCI on follicular genesis and histologic features of ovarian follicles in PCOS rats. Relative gene expression related to follicular genesis involving KIT ligand (*Kitl*) and hyaluronan synthase 2 (*Has2*) as assessed by quantitative Real-Time PCR. *Rplp0* was used as an internal control. All values represent mean ±SD. ^a^ *p* < 0.05 vs. vehicle, ^b^ *p*< 0.05 vs. PCOS, ^c^ *p*< 0.05 vs. PCOS + DCI; ^d^ *p*< 0.05 vs. PCOS + EC. MET, metformin; EC, *Ecklonia cava* K.; DCI, *d-chiro* inositol.

To evaluate the follicle formation effect of EC and DCI, the mRNA levels of the genes Kitl and Has2, which are related to folliculogenesis, were analyzed. The Kitl mRNA expression level was reduced in the group treated with letrozole and was elevated in the group cotreated with DCI and EC (Figure 5). Parallel to the Kitl mRNA expression level, the Has mRNA expression level was also lowered in the PCOS group and was shown to be significantly increased compared to the PCOS group by EC and/or DCI treatment, as shown in Figure 5. The ratio of cystic follicles to mature follicles was not the same among the groups but was recovered to the normal group ratio by EC and/or DCI (Figure 5). The thicknesses of the theca cell and granulosa cell layers were also not the same among the groups but were returned to the normal group level by EC and/or DCI (Figure 5).

### Histological Features and Localization of Aromatase

**FIGURE 6:**
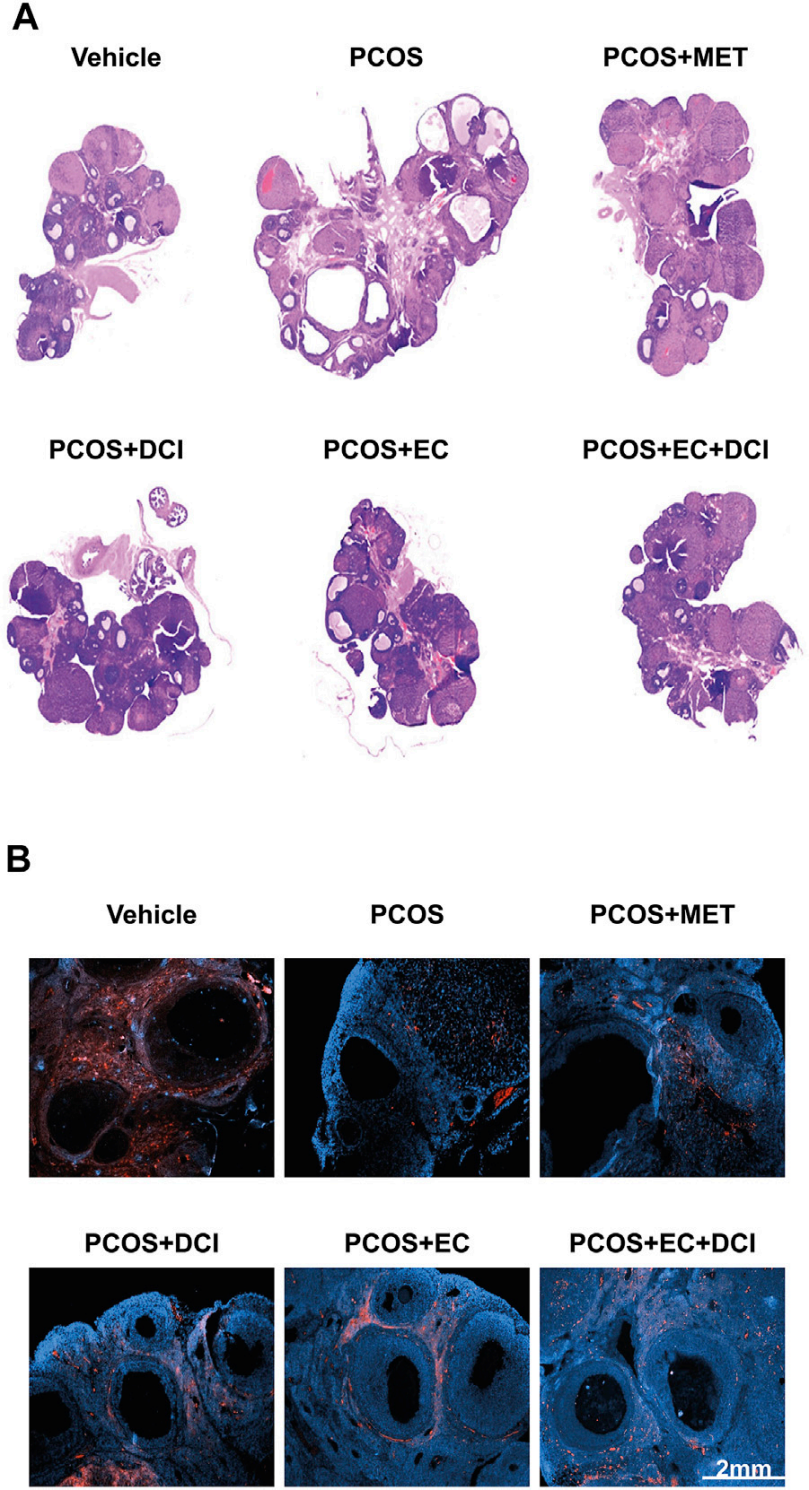
Effect of EC extract and DCI on aromatase localization and histological changes in the ovary of PCOS rats. **(A)** H&E-stained ovarian tissue was scanned with a slide scanner (3D HISTECH Ltd., Budapest, Hungary). Magnification ×20. Section of ovary was performed immunohistochemistry using specific aromatase **(B)** antibody; 2nd Ab was rabbit (Scale bar = 2 mm), counterstained with DAPI. Magnification ×200.

To evaluate the effectiveness of EC and DCI in PCOS rat ovarian tissue, hematoxylin and eosin (H&E) staining was performed on ovarian tissue and analyzed under a microscope. Overall, cystic follicles appeared frequently in the PCOS group, and in contrast, mature follicles were hardly observed (Figure 6). On the other hand, cystic follicles were rarely observed in the group treated with EC and DCI compared to the PCOS group, and the appearance of mature follicles also became more frequent. The location and expression values of aromatase were confirmed using immunohistochemical staining. Aromatase was abundantly expressed in the layer of follicular cells in the normal group, whereas this expression was not readily abundant in the follicular cells in the PCOS group (Figure 6).

### Effects of *Ecklonia cava* K. and D-Chiro-Inositol on Hormone Receptors and Inflammatory Cytokine-Related Genes

**FIGURE 7:**
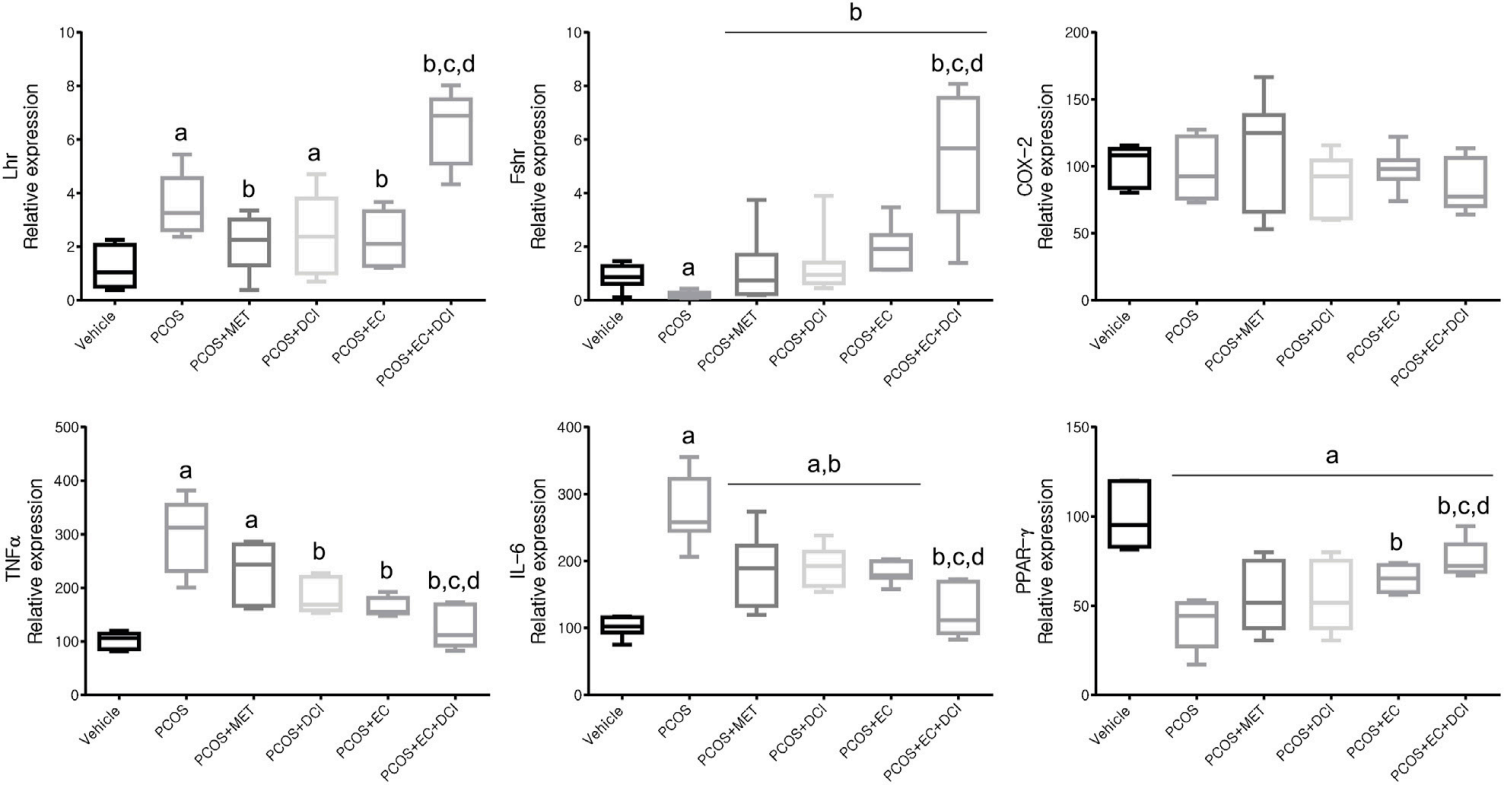
Effect of EC extract and DCI on ovarian gene expression related to hormone receptors and inflammation in PCOS rats. Compared mRNA expression levels of *Lhr*, *Fshr*, COX-2, TNFα, IL-6, and PPARγ from the ovaries of each experimental group as assessed by quantitative Real-Time PCR. *Rplp0* was used as an internal control gene. All values represent means ±SD. ^a^ *p* < 0.05 vs. vehicle, ^b^ *p*< 0.05 vs. PCOS, ^c^ *p*< 0.05 vs. PCOS + DCI, ^d^ *p*< 0.05 vs. PCOS + EC. MET, metformin; EC, *Ecklonia cava* K.; DCI, *d-chiro* inositol.

To estimate the transcription level of hormone receptor-related genes, the mRNA expression levels of LHR and FSHR were investigated. The mRNA expression levels of inflammatory cytokines in ovarian tissue were also evaluated (Figure 7). The LHR mRNA expression level was the highest in the group treated with EC and DCI, and FSHR mRNA expression was similar to that of LHR in the ovary. Inflammatory cytokine, including TNFα and IL-6, mRNA expression levels were all significantly increased in the PCOS group and tended to decrease effectively in the EC and/or DCI treatment groups (Figure 7). On the other hand, anti-inflammatory cytokine and PPAR-γ mRNA expression decreased in the PCOS group, but not in parallel with the expression of inflammatory cytokines such as TNFα and IL-6, and were restored to normal group levels by EC and DCI treatment.

## Discussion

Women with healthy reproductive cycles experience menstruation every month after the first ovulation. This is essential for women’s health, representing normal steroid production, which systematically affects reproductive organ development, endocrine interactions, and mood disorders (Freeman et al., 2006). In women with PCOS, after failure of ovulation, stimulation of ovulation is still considered the first-line medical approach to improving fertility (Palomba et al., 2004). In women with anovulatory infertility, the first treatment option for induction of ovulation is clomiphene citrate, the most common (Kar, 2012; Tripathy et al., 2013). However, one-fourth of patients are resistant to clomiphene citrate and do not ovulate (Tripathy et al., 2013). In some cases, compared to the first treatment option available in previous studies, it is more effective in women with PCOS when administered together with ovulation-inducing agents and other drugs (Wang et al., 2017).

In this study, the effects of EC extract and/or DCI in a rat model of PCOS-like symptoms were evaluated. There was a significant difference between sham-operated and letrozole-treated rats in terms of body weight. However, no significant weight change was observed between the PCOS group and the EC extract and/or DCI treatment groups. On the other hand, a weight change in peritoneal posterior fat was observed, and the fat weight increased in the PCOS group and decreased in the metformin-treated group and the two groups treated with EC extract (PCOS + EC and PCOS + EC + DCI). There was no difference in uterine weight, blood sugar, or insulin concentrations among the experimental groups, but the emergence of vaginal smear cells was observed in the group that was administered both compounds together compared to the groups that were administered DCI or EC alone. This result means that arrest in the estrous cycle is alleviated, and changes in blood ovarian hormone concentrations can be inferred. An earlier study showed that in a mouse model, the histopathological and functional characteristics of PCOS were almost completely reversed by treating animals with the inositol complex (Bevilacqua et al., 2019). As phosphorylated derivatives, Ml and DCI are the second-largest insulin messengers, but their regulation has specific individual roles (Asplin et al., 1993). In addition, it is known that the MI:DCI content affects the epimerase activity and insulin resistance of ovarian tissue in patients with PCOS, and short-term DCI treatments successfully induce ovulation in women with PCOS (Nestler et al., 1999).

At the blood hormone level, the levels of LH and T increased in the PCOS group, while the concentration of E2 decreased. In addition, the increased concentrations of LH and T tended to be more pronounced in the groups administered EC and DCI together than in the EC and DCI single administration groups. The concentration of E2 in the blood was observed to be the lowest in the PCOS group, and although it fluctuated in the groups treated with EC and DCI, there was no significance. A decrease in the blood concentrations of LH and T is used as an important indicator when determining symptom relief of PCOS and is considered a stage in the restoration of normal ovaries and uterine cycles (Goldman et al., 2007).

The concentrations of TNFα and IL-6 in the blood were observed to be high in the PCOS group, and the decrease was observed to be the largest in the EC and DCI groups. In addition, transcriptional expression of TNFα and IL-6 in ovarian tissue increased in the PCOS group, and their levels were similar to the tendencies of their blood concentrations. The expression of PPAR-γ was lowered in the PCOS group and was increased by EC and/or DCI.

In a previous study, it was known that the imbalance of anti-inflammatory cytokines was closely related to insulin resistance and may play a role in the development of PCOS (Elkind-Hirsch, 2006). TNFα and IL-6 are important proinflammatory cytokines that can affect ovarian function (Kim et al., 2011). Previous studies have shown that the concentrations of TNFα and IL-6 were higher in the blood and follicular fluid of infertile women with PCOS than in the control group (Amato et al., 2003; Kim et al., 2011). PPAR-γ has been shown to control the unique network of downstream genes, including those encoding TNFα and IL-6. There is also an opinion that chronic inflammation caused by changes in TNFα and IL-6 may have a detrimental effect on fertilization (Chinetti et al., 2000; Lee et al., 2017).

In ovarian tissue, the transcriptional expression of LHR and FSHR showed the highest expression in the EC and DCI cotreated groups compared to the other experimental groups. In addition, the transcriptional expression of Kitl and Has2 was also observed to be the lowest in the PCOS group, with the highest expression tendency in the EC and DCI cotreatment groups. Previously, FSHR has been shown to exert synergies with other stimulators, such as androgen, to moderately regulate follicular growth in the basal follicle growth phase, and LHR is also abundantly expressed on the surfaces of theca cells and affects ovulation, corpus luteum formation and ovarian hormone stimulation (Edson et al., 2009). Kitl is sufficient and essential to induce primitive follicle development, and the expansion of the cumulus requires the secretion of FSH and epidermal growth factor (EGF), which is also associated with the elevation of Has2 mRNA (Camaioni et al., 1993). COX2 is produced by cumulus cells and is necessary for maximum cumulus expansion and ovulation (Lim et al., 1997).

We also analyzed the ovaries of rats by H&E staining and measured protein expression and the location of aromatase using specific antibodies. In the PCOS group, the layer of granulosa cells was thinner than that in the normal group, whereas the layer of theca cells was thicker. In addition, the change was restored in the group that was treated with EC and DCI. In the PCOS group, the number of cystic follicles increased, and the number of mature follicles decreased. These changes were also normalized by EC and DCI treatment.

All of our experimental results suggest improvement in ovarian function progression upon EC extract and/or DCI treatment in PCOS rats. EC and/or DCI promoted aromatase performance, relieved the blocked conversion of T into E2, and restored hormonal balance, thereby affecting ovarian morphology. Furthermore, EC and DCI were involved in the remedy of ovarian dysfunction by controlling the levels of inflammatory cytokines in the blood and ovarian tissue. The improvement with the cotreatment of EC and DCI was more effective than that of a single treatment, which may be utilized as a basis for novel PCOS therapeutic methods in PCOS women.

## Data Availability

The raw data supporting the conclusion of this article will be made available by the authors without undue reservation.

## References

[B1] Amato G. Conte M. Mazziotti G. Lalli E. Vitolo G. Tucker A. T. (2003). Serum and Follicular Fluid Cytokines in Polycystic Ovary Syndrome during Stimulated Cycles. Obstet. Gynecol. 101 (6), 1177–1182. 10.1016/s0029-7844(03)00233-3 12798522

[B2] Asplin I. Galasko G. Larner J. (1993). Chiro-inositol Deficiency and Insulin Resistance: A Comparison of the Chiro-Inositol- and the Myo-Inositol-Containing Insulin Mediators Isolated from Urine, Hemodialysate, and Muscle of Control and Type Ii Diabetic Subjects. Proc. Natl. Acad. Sci. U. S. A. 90 (13), 5924–5928. 10.1073/pnas.90.13.5924 8392181 PMC46839

[B3] Bashir S. T. Baerwald A. R. Gastal M. O. Pierson R. A. Gastal E. L. (2018). Follicle Growth and Endocrine Dynamics in Women with Spontaneous Luteinized Unruptured Follicles versus Ovulation. Hum. Reprod. 33 (6), 1130–1140. 10.1093/humrep/dey082 29659847

[B4] Bates G. W. Legro R. S. (2013). Longterm Management of Polycystic Ovarian Syndrome (Pcos). Mol. Cell. Endocrinol. 373 (1-2), 91–97. 10.1016/j.mce.2012.10.029 23261983 PMC4367484

[B5] Bevilacqua A. Dragotto J. Giuliani A. Bizzarri M. (2019). Myo-inositol and D-Chiro-Inositol (40:1) Reverse Histological and Functional Features of Polycystic Ovary Syndrome in a Mouse Model. J. Cell. Physiol. 234 (6), 9387–9398. 10.1002/jcp.27623 30317628

[B6] Bremer A. A. (2010). Polycystic Ovary Syndrome in the Pediatric Population. Metab. Syndr. Relat. Disord. 8 (5), 375–394. 10.1089/met.2010.0039 20939704 PMC3125559

[B7] Camaioni A. Hascall V. C. Yanagishita M. Salustri A. (1993). Effects of Exogenous Hyaluronic Acid and Serum on Matrix Organization and Stability in the Mouse Cumulus Cell-Oocyte Complex. J. Biol. Chem. 268 (27), 20473–20481. 10.1016/s0021-9258(20)80750-9 8376402

[B8] Chinetti G. Fruchart J. C. Staels B. (2000). Peroxisome Proliferator-Activated Receptors (Ppars): Nuclear Receptors at the Crossroads between Lipid Metabolism and Inflammation. Inflamm. Res. 49 (10), 497–505. 10.1007/s000110050622 11089900

[B9] Dăneasă A. Cucolaş C. Lenghel L. M. Olteanu D. Orăsan R. G. A. Filip G. A. (2016). Letrozole vs Estradiol Valerate Induced Pcos in Rats: Glycemic, Oxidative and Inflammatory Status Assessment. Reproduction 151 (4), 401–409. 10.1530/REP-15-0352 26792865

[B10] Donguibogam-Committee (1999). Translated Donguibogam. Seoul, Republic of Korea: Bubinmunwha Press.

[B11] Edson M. A. Nagaraja A. K. Matzuk M. M. (2009). The Mammalian Ovary from Genesis to Revelation. Endocr. Rev. 30 (6), 624–712. 10.1210/er.2009-0012 19776209 PMC2761115

[B12] Elkind-Hirsch K. E. (2006). Thiazolidinediones for the Therapeutic Management of Polycystic Ovary Syndrome : Impact on Metabolic and Reproductive Abnormalities. Treat. Endocrinol. 5 (3), 171–187. 10.2165/00024677-200605030-00005 16677059

[B13] Falorni A. Minarelli V. Eads C. M. Joachim C. M. Persani L. Rossetti R. (2014). A Clinical Research Integration Special Program (Crisp) for Young Women with Primary Ovarian Insufficiency. Panminerva Med. 56 (4), 245–261. Available at: https://pubmed.ncbi.nlm.nih.gov/25288327/ 25288327 PMC4532281

[B14] Freeman E. W. Sammel M. D. Lin H. Nelson D. B. (2006). Associations of Hormones and Menopausal Status with Depressed Mood in Women with No History of Depression. Arch. Gen. Psychiatry 63 (4), 375–382. 10.1001/archpsyc.63.4.375 16585466

[B15] Goldman J. M. Murr A. S. Cooper R. L. (2007). The Rodent Estrous Cycle: Characterization of Vaginal Cytology and its Utility in Toxicological Studies. Birth Defects Res. B Dev. Reprod. Toxicol. 80 (2), 84–97. 10.1002/bdrb.20106 17342777

[B16] Kar S. (2012). Clomiphene Citrate or Letrozole as First-Line Ovulation Induction Drug in Infertile Pcos Women: A Prospective Randomized Trial. J. Hum. Reprod. Sci. 5 (3), 262–265. 10.4103/0974-1208.106338 23531705 PMC3604833

[B17] Kauffman A. S. Thackray V. G. Ryan G. E. Tolson K. P. Glidewell-Kenney C. A. Semaan S. J. (2015). A Novel Letrozole Model Recapitulates Both the Reproductive and Metabolic Phenotypes of Polycystic Ovary Syndrome in Female Mice. Biol. Reprod. 93 (3), 69. 10.1095/biolreprod.115.131631 26203175 PMC4710190

[B18] Kim C. H. Ahn J. W. You R. M. Kim S. H. Chae H. D. Kang B. M. (2011). Pioglitazone Treatment Decreases Follicular Fluid Levels of Tumor Necrosis Factor-α and Interleukin-6 in Patients with Polycystic Ovary Syndrome. Clin. Exp. Reprod. Med. 38 (2), 98–102. 10.5653/cerm.2011.38.2.98 22384426 PMC3283053

[B19] Kim S. Choi S. I. Kim G. H. Imm J. Y. (2019). Anti-inflammatory Effect of Ecklonia Cava Extract on Porphyromonas Gingivalis Lipopolysaccharide-Stimulated Macrophages and a Periodontitis Rat Model. Nutrients 11 (5), 1143. 10.3390/nu11051143 PMC6566535 31121899

[B20] Laganà A. S. Unfer V. (2019). D-chiro-inositol's Action as Aromatase Inhibitor: Rationale and Potential Clinical Targets. Eur. Rev. Med. Pharmacol. Sci. 23 (24), 10575–10576. 10.26355/eurrev_201912_19752 31858524

[B21] Lee J. Y. Tae J. C. Kim C. H. Hwang D. Kim K. C. Suh C. S. (2017). Expression of the Genes for Peroxisome Proliferator-Activated Receptor-γ, Cyclooxygenase-2, and Proinflammatory Cytokines in Granulosa Cells from Women with Polycystic Ovary Syndrome. Clin. Exp. Reprod. Med. 44 (3), 146–151. 10.5653/cerm.2017.44.3.146 29026721 PMC5636927

[B22] Lim H. Paria B. C. Das S. K. Dinchuk J. E. Langenbach R. Trzaskos J. M. (1997). Multiple Female Reproductive Failures in Cyclooxygenase 2-deficient Mice. Cell. 91 (2), 197–208. 10.1016/s0092-8674(00)80402-x 9346237

[B23] Nestler J. E. Jakubowicz D. J. Reamer P. Gunn R. D. Allan G. (1999). Ovulatory and Metabolic Effects of D-Chiro-Inositol in the Polycystic Ovary Syndrome. N. Engl. J. Med. 340 (17), 1314–1320. 10.1056/NEJM199904293401703 10219066

[B24] Palomba S. Orio F. Jr. Russo T. Falbo A. Cascella T. Colao A. (2004). Is Ovulation Induction Still a Therapeutic Problem in Patients with Polycystic Ovary Syndrome? J. Endocrinol. Invest. 27 (8), 796–805. 10.1007/BF03347527 15636438

[B25] Rausch M. E. Legro R. S. Barnhart H. X. Schlaff W. D. Carr B. R. Diamond M. P. (2009). Predictors of Pregnancy in Women with Polycystic Ovary Syndrome. J. Clin. Endocrinol. Metab. 94 (9), 3458–3466. 10.1210/jc.2009-0545 19509098 PMC2741722

[B26] Sahmay S. Usta T. A. Erel T. Atakul N. Aydogan B. (2014). Elevated Lh Levels Draw a Stronger Distinction Than Amh in Premature Ovarian Insufficiency. Climacteric 17 (2), 197–203. 10.3109/13697137.2013.870149 24299186

[B27] Shi D. Vine D. F. (2012). Animal Models of Polycystic Ovary Syndrome: A Focused Review of Rodent Models in Relationship to Clinical Phenotypes and Cardiometabolic Risk. Fertil. Steril. 98 (1), 185–193. 10.1016/j.fertnstert.2012.04.006 22607890

[B28] Tripathy S. Mohapatra S. Muthulakshmi M. Chandrasekhar A. (2013). Induction of Ovulation with Clomiphene Citrate versus Clomiphene with Bromocriptine in Pcos Patients with Normal Prolactin: A Comparative Study. J. Clin. Diagn Res. 7 (11), 2541–2543. 10.7860/JCDR/2013/7617.3605 24392395 PMC3879865

[B29] Trivax B. Azziz R. (2007). Diagnosis of Polycystic Ovary Syndrome. Clin. Obstet. Gynecol. 50 (1), 168–177. 10.1097/GRF.0b013e31802f351b 17304034

[B30] Unfer V. Orrù B. G. Monastra G. (2016). Inositols: From Physiology to Rational Therapy in Gynecological Clinical Practice. Expert Opin. Drug Metab. Toxicol. 12 (10), 1129–1131. 10.1080/17425255.2016.1225039 27564226

[B31] Wang R. Kim B. V. van Wely M. Johnson N. P. Costello M. F. Zhang H. (2017). Treatment Strategies for Women with Who Group Ii Anovulation: Systematic Review and Network Meta-Analysis. BMJ 356, j138. 10.1136/bmj.j138 28143834 PMC5421445

[B32] Yang H. Lee S. Y. Lee S. R. Pyun B. J. Kim H. J. Lee Y. H. (2018). Therapeutic Effect of Ecklonia Cava Extract in Letrozole-Induced Polycystic Ovary Syndrome Rats. Front. Pharmacol. 9, 1325. 10.3389/fphar.2018.01325 30524282 PMC6262357

